# Evaluation of Attention-Deficit/Hyperactivity Disorder Medications, Externalizing Symptoms, and Suicidality in Children

**DOI:** 10.1001/jamanetworkopen.2021.11342

**Published:** 2021-06-04

**Authors:** Gal Shoval, Elina Visoki, Tyler M. Moore, Grace E. DiDomenico, Stirling T. Argabright, Nicholas J. Huffnagle, Aaron F. Alexander-Bloch, Rebecca Waller, Luke Keele, Tami D. Benton, Raquel E. Gur, Ran Barzilay

**Affiliations:** 1 Geha Mental Health Center and Sackler School of Medicine, Tel Aviv University, Tel Aviv, Israel; 2 Princeton Neuroscience Institute, Princeton University, Princeton, New Jersey; 3 Department of Child and Adolescent Psychiatry and Behavioral Science, Children’s Hospital of Philadelphia, Philadelphia, Pennsylvania; 4 Lifespan Brain Institute of Children’s Hospital of Philadelphia and Penn Medicine, Philadelphia, Pennsylvania; 5 Department of Psychiatry, Perelman School of Medicine, University of Pennsylvania, Philadelphia; 6 Department of Psychology, University of Pennsylvania, Philadelphia; 7 Division of Epidemiology and Biostatistics, Department of Surgery, Perelman School of Medicine, University of Pennsylvania, Philadelphia

## Abstract

**Importance:**

Childhood suicidality (ie, suicidal ideation or attempts) rates are increasing, and attention-deficit/hyperactivity disorder (ADHD) and externalizing symptoms are common risk factors associated with suicidality. More data are needed to describe associations of ADHD pharmacotherapy with childhood suicidality.

**Objective:**

To investigate the associations of ADHD pharmacotherapy with externalizing symptoms and childhood suicidality.

**Design, Setting, and Participants:**

In this cohort study, cross-sectional and 1-year-longitudinal associations were examined using data (collected during 2016-2019) from the Adolescent Brain Cognitive Development (ABCD) Study, a large, diverse US sample of children aged 9 to 11 years. Data analysis was performed from November to December 2020.

**Exposures:**

Main and interaction associations of externalizing symptoms (hyperactivity ADHD symptoms, oppositional defiant, and conduct disorder symptoms) and ADHD medication treatment (methylphenidate and amphetamine derivatives, α-2-agonists, and atomoxetine) at baseline assessment.

**Main Outcomes and Measures:**

Child-reported suicidality (past and present at baseline; current at longitudinal assessment). Covariates were age, sex, race/ethnicity, parents’ education, marital status, and concomitant child psychiatric pharmacotherapy (antidepressants and antipsychotics).

**Results:**

Among 11 878 children at baseline assessment (mean [SD] age, 9.9 [0.6] years; 6196 boys [52.2%]; 8805 White [74.1%]), 1006 (8.5%) were treated with ADHD medication and 1040 (8.8%) reported past or current suicidality. Externalizing symptoms (median [range], 1 [0-29] symptom count) were associated with suicidality (for a change of 1 SD in symptoms, odds ratio [OR], 1.34; 95% CI, 1.26-1.42; *P* < .001), as was ADHD medication treatment (OR, 1.32; 95% CI, 1.06-1.64; *P* = .01). ADHD medication use was associated with less suicidality in children with more externalizing symptoms (significant symptom-by-medication interaction, B = −0.250; SE = 0.086; *P* = .004), such that for children who were not receiving ADHD medications, there was an association between more externalizing symptoms and suicidality (for a change of 1 SD in symptoms, OR, 1.42; 95% CI, 1.33-1.52; *P* < .001); however, for children who were receiving ADHD medication, there was no such association (OR, 1.15; 95% CI, 0.97-1.35; *P* = .10). The association with medication remained even when covarying for multiple confounders, including risk and protective factors for suicidality in ABCD, and was replicated in 1-year longitudinal follow-up. Sensitivity analyses matching participants with high numbers of externalizing symptoms taking and not taking ADHD medication treatment confirmed its association with less suicidality.

**Conclusions and Relevance:**

These findings suggest that ADHD medication treatment is associated with less suicidality in children with substantial externalizing symptoms and may be used to inform childhood suicide prevention strategies.

## Key Points

**Question:**

Are there associations among attention-deficit/hyperactivity disorder (ADHD) pharmacotherapy, externalizing symptoms, and childhood suicidality (ie, ideation or attempts)?

**Findings:**

In this cohort study of 11 878 children aged 9 to 10 years at baseline, externalizing symptoms showed expected associations with suicidality. ADHD pharmacotherapy was associated with less suicidality in children with externalizing symptoms, specifically among those with substantial symptoms burden, both at the baseline and 1-year follow-up assessment.

**Meaning:**

These findings suggest that in children with substantial externalizing symptoms, ADHD medication use may be associated with less suicidality, in addition to its known clinical benefits in treating externalizing symptoms.

## Teaser

This cohort study investigates the associations of attention-deficit/hyperactivity disorder (ADHD) medication with externalizing symptoms and childhood suicidality.

## Introduction

Suicidal behavior is a major public health concern in children worldwide,^1^ with steadily increasing rates.^2,3,4,5,6,7^ Thus, there is a critical need to identify modifiable risk and protective factors.^8^ Attention-deficit/hyperactivity disorder (ADHD) is a common neurodevelopmental disorder typically diagnosed during childhood, with worldwide prevalence rates in school-age children of 5% to 7%.^9,10^ ADHD is associated with multiple psychiatric comorbidities,^11,12,13,14^ including suicidal ideation, suicide attempts, and completed suicide, even after controlling for other comorbid mental health disorders.^15,16,17^ Similarly, other externalizing disorders (eg, oppositional defiant disorder [ODD] and conduct disorder [CD]) are factors associated with risk of suicidal behavior,^18,19,20^ specifically during the elementary school years.^21,22,23^

Recent studies^24,25,26,27^ have examined the association of ADHD pharmacotherapy with suicidal behavior. Large-scale population-based studies^24,25,26,27^ from Sweden, Taiwan, and the US indicated that psychostimulants, particularly methylphenidate, may be associated with less suicidal behavior in patients with ADHD. However, these studies relied on national registries or insurance claims with limited granularity in clinical phenotypes, including symptom severity and other confounders that are associated with medication treatment and suicidality, such as socioeconomic status and home and school environment.^27^ Thus, more data are needed on the potential relevance of ADHD medications use in children, which is increasing globally,^28,29^ to suicide prevention.

Our main objective was to determine the association of ADHD pharmacotherapy with child suicidal ideation or attempts (ie, suicidality) in a large, diverse longitudinal cohort from the Adolescent Brain Cognitive Development (ABCD) Study (11 878 children recruited at ages 9-10 years).^30^ Our hypotheses were that externalizing symptoms and ADHD medications would be independently associated with higher likelihood of reporting suicidality at baseline and at 1-year follow-up. Furthermore, because ADHD medications are established as effective for treating externalizing (ADHD-related) symptoms, we hypothesized an interaction, such that ADHD pharmacotherapy would moderate the association between externalizing symptoms and risk of suicidality both at baseline and 1-year follow-up assessments (ie, externalizing symptoms by medication interaction).

## Methods

### Participants

The ABCD Study sample includes a cohort of 11 878 children aged 9 to 10 years at baseline, recruited through school systems.^31^ Participants were enrolled at 21 sites, with a catchment area encompassing more than 20% of the entire US population in this age group. We included data from the ABCD Study data release 3.0. All included measures in the current analyses were collected at baseline assessment, with 2 variables collected at 1-year follow-up (age and current suicidality). All participants gave assent. Parents and/or caregivers provided written informed consent. The ABCD Study protocol was approved by the University of California, San Diego, institutional review board and was exempted from a full review by University of Pennsylvania institutional review board. This study follows the Strengthening the Reporting of Observational Studies in Epidemiology (STROBE) reporting guideline for reporting observational studies.

### Exposures

Child externalizing symptom count at baseline assessment (reported as past or current) was based on the parent and/or caregiver–validated and computerized Kiddie-Structured Assessment for Affective Disorders and Schizophrenia for *Diagnostic and Statistical Manual of Mental Disorders* (Fifth Edition) (KSADS-5)^32^ and included ADHD hyperactivity symptoms, ODD symptoms, and CD symptoms (eTable 1 in the Supplement). Data on prescribed medication at baseline assessment were collected from caregivers using the Medication Inventory from the PhenX instrument.^33^ Caregivers were asked to bring the youth’s prescription medication (used in the last 2 weeks) to the in-person assessment.^30^ We considered any of the following classes of medication as ADHD medications: methylphenidate derivatives, amphetamine derivatives, α-2-agonists, and atomoxetine (eTable 2 in the Supplement).

### Outcome Measures

The KSADS-5 assessed suicidal ideation and attempt (past or current).^34^ Items relating to self-injurious behavior without suicidal intent were not included. Because the proportion of suicidal attempts was low, and to mitigate risk of type I error caused by multiple testing, we combined suicidal ideation and attempt, consistent with previous analyses.^34^ Thus, suicidal outcomes in the baseline assessment (past or current) were collapsed into a single binary measure termed *suicidality*. Prevalence of suicidal ideation and attempts is detailed in eTable 3 in the Supplement. In the follow-up assessment, we referred only to current suicidality (past 2 weeks) to establish a temporal association between baseline and 1-year follow-up assessment. Because prior studies^35,36,37^ (including in the ABCD Study) showed poor youth-caregiver agreement on suicidality, we focused on child-report in the main analyses.

### Covariates

To address the diversity of the ABCD Study population, models included age, sex, race (self-reported as White, Black, Asian, or other, which included American Indian, Native Hawaiian, and a category reported by participants as *other*), Hispanic ethnicity, parent education (mean duration), and marital status (married, divorced or separated, or other). To address confounding associations with other psychiatric medications, we included a binary variable indicating treatment with any antidepressant (AD) or antipsychotic (AP) medication (eTable 2 in the Supplement). To address confounding effects of other variables that were previously associated with childhood suicidality in the ABCD Study,^34^ we ran models covarying for family conflict, weekend screen time, parental supervision, and school involvement. To address confounding associations of comorbid depression and/or anxiety, we created 2 binary variables (for depression and for anxiety) using KSADS-5–derived diagnoses (eTable 4 in the Supplement).

### Statistical Analysis

The analytical plan and hypotheses were preregistered on Open Science Framework on October 2020, and analyses were conducted in November to December 2020 following the ABCD Study data release 3.0, which was the first release of 1-year suicidality measures for the entire sample. We used the SPSS statistical package version 26.0 (IBM) and R statistical software version 3.6.1 (R Project for Statistical Computing) for our data analyses. Mean (SD) and frequency (percentage) were reported for descriptive purposes. Univariable comparisons were made using *t* tests or χ^2^ tests, as appropriate. Statistical significance was set at *P* < .05. We used listwise deletion for participants with missing data (<1.27% for baseline data and 6.74% for 1-year follow-up data).

#### Main Analysis

To address the study’s question of the moderating association of ADHD medications on the association between externalizing symptoms and suicidality (referred to later as main models), we performed binary logistic regression where the dependent variable was baseline child-reported suicidality (cross-sectional model) or current 1-year follow-up suicidality (longitudinal model), and the independent variable of interest was the interaction term of externalizing symptoms (*z* score of sum) and ADHD medication (binary variable). To allow easier interpretation of main associations, we first tested the main associations of externalizing symptoms and ADHD medication without the interaction term. ADHD medication use was regressed out of the symptom count to address collinearity (ie, with higher externalizing symptoms the chance of receiving ADHD medication increases). We also ran a model with externalizing symptoms as the independent variable separately in children taking or not taking ADHD medication. Covariates were age, sex, race/ethnicity, parents’ education and marital status, and receipt of other psychiatric medications (AD or AP). The longitudinal model also included time between assessments (in months) and baseline suicidality. We used 2-tailed tests for all cross-sectional models and 1-tailed tests in confirmatory longitudinal models and case-matched sensitivity analyses.

#### Sensitivity Analyses

We conducted several sensitivity analyses (detailed in eMethods in the Supplement). In brief, to evaluate robustness of results, we covaried for established risk and protective factors for suicidality previously described in the ABCD Study.^34^ To account for potential bias following our choice of exposures or dependent variables, we ran the main models using different definitions of externalizing symptoms and of suicidality. Additional sensitivity analyses were done using an imputed data set (created using the R library Amelia) and addressing potential associations with family relatedness. Finally, to better estimate clinical presentations where ADHD medication is especially associated with less suicidality, we matched participants receiving ADHD medications with high externalizing symptom loads at 3 thresholds (>1, >2, or >3 SDs) with controls not receiving ADHD medications and compared suicidality rates.

#### Exploratory Analyses

Exploratory analyses were conducted to evaluate the association of sex and the association of receiving any AD or AP medication with the main study question (described in detail in eMethods in the Supplement). To explore potential differences in the moderating associations of different ADHD medication classes on the association between externalizing symptoms and suicidality, we tested 4 additional models, 1 for each medication class.

## Results

### Association of Externalizing Symptoms and ADHD Medications With Suicidality at Baseline Assessment

Among 11 878 children at the baseline ABCD Study assessment (mean [SD] age, 9.9 [0.6] years; 6196 boys [52.2%]; 8805 White [74.1%]), 1006 (8.5%) were treated with ADHD medication and 1040 (8.8%) reported past or current suicidality (Table 1). More boys than girls reported suicidality (603 boys [58.0%] vs 437 girls [42.0%]), with no differences in race, ethnicity, or parents’ education. Children reporting suicidality had more externalizing symptoms (mean [SD] symptoms, 6.6 [6.7] vs 4.1 [5.6]; median [range], 1 [0-29] symptoms) and higher prevalence of ADHD medication use compared with nonsuicidal children (136 [13.1%] vs 863 [8.0%] children), as well as more AD (65 [6.3%] vs 158 [1.5%] children) and AP (20 [1.9%] vs 50 [0.5%] children) medication use (Table 1).

**Table 1.** Sample Sociodemographic and Clinical Characteristics

Characteristic	Baseline, participants, No. (%)			*P* value^c^	1-y Follow-up, participants, No. (%)^a^		*P* value^c^
	Total sample (n = 11 878)^**b**^	Suicidality (n = 1040)	Control (n = 10 764)		Current suicidality (n = 198)	Control (n = 10 879)	
Age at assessment, mean (SD), y	9.9 (0.6)	9.9 (0.6)	9.9 (0.6)	.60	10.9 (0.6)	10.9 (0.6)	.51
Sex							
Male	6196 (52.2)	603 (58.0)	5559 (51.6)	<.001	96 (48.5)	5702 (52.4)	.27
Female	5682 (47.8)	437 (42.0)	5205 (48.4)		102 (51.2)	5177 (47.6)	
Race/ethnicity							
White	8805 (74.1)	759 (73.0)	7995 (74.3)	.36	129 (65.2)	8219 (75.5)	.001
Black	2518 (21.2)	239 (23.0)	2261 (21.0)	.14	62 (31.3)	2174 (20.0)	<.001
Asian	752 (6.3)	69 (6.6)	676 (6.3)	.65	12 (6.1)	700 (6.4)	.83
Hispanic	2411 (20.3)	196 (19.1)	2196 (20.7)	.24	49 (25.1)	2145 (20.0)	.07
Parents’ education, mean (SD), y	16.4 (2.7)	16.4 (2.6)	16.4 (2.7)	.90	15.9 (3.0)	16.5 (2.7)	.003
Parents married	7991 (66.9)	649 (62.4)	7297 (67.8)	<.001	116 (58.6)	7474 (68.7)	.003
Parents divorced or separated	1546 (13.0)	166 (16.0)	1371 (12.7)	.003	31 (15.7)	1381 (12.7)	.21
Externalizing symptoms, mean (SD), No.	4.3 (5.8)	6.6 (6.7)	4.1 (5.6)	<.001	6.3 (6.7)	4.2 (5.7)	<.001
Any externalizing diagnosis	3255 (27.4)	430 (41.7)	2812 (26.4)	<.001	74 (38.1)	2936 (27.3)	<.001
ADHD diagnosis	2550 (25.5)	341 (33.1)	2198 (20.6)	<.001	60 (30.9)	2298 (21.4)	.001
Oppositional defiance disorder diagnosis	1667 (14.0)	245 (23.8)	1418 (13.3)	<.001	40 (20.6)	1491 (13.9)	.007
Conduct disorder diagnosis	375 (3.2)	66 (6.4)	306 (2.9)	<.001	13 (6.7)	320 (3.0)	.003
ADHD medications							
Any	1006 (8.5)	136 (13.1)	863 (8.0)	<.001	28 (14.1)	912 (8.4)	.004
Methylphenidate	541 (4.6)	72 (6.9)	467 (4.3)	<.001	17 (8.6)	495 (4.6)	.007
Amphetamine	363 (3.1)	43 (4.1)	317 (2.9)	.03	6 (3.0)	324 (3.0)	.97
α-2-Agonists	243 (2.0)	35 (3.4)	205 (1.9)	.001	5 (2.5)	217 (2.0)	.60
Atomoxetine	47 (0.4)	10 (1.0)	37 (0.3)	.002	3 (1.5)	43 (0.4)	.02
Other psychiatric medications							
Any	273 (2.3)	77 (7.4)	194 (1.8)	<.001	8 (4.0)	246 (2.3)	.10
Antidepressants	224 (1.9)	65 (6.3)	158 (1.5)	<.001	7 (3.5)	203 (1.9)	.09
Antipsychotics	71 (0.6)	20 (1.9)	50 (0.5)	<.001	1 (0.5)	64 (0.6)	.88

Abbreviation: ADHD, attention-deficit/hyperactivity disorder.

^a^ Follow-up data were available for 11 077 participants (6.74% missing data of the baseline sample).

^b^ For all variables, missing data rate was less than 1.27% (151 participants of the 11 878 participants at baseline Adolescent Brain Cognitive Development Study assessment).

^c^ *P* values were calculated with *t* test and χ^2^ test for continuous and binary measures, respectively.

Multivariable logistic regression models revealed main associations of externalizing symptoms (for a change of 1 SD in externalizing symptoms, odds ratio [OR], 1.34; 95% CI 1.26-1.42; *P* < .001) and ADHD medication (OR, 1.32; 95% CI, 1.06-1.64; *P* = .01) with suicidality, covarying for demographic characteristics and use of other psychiatric medications (AD or AP). These main associations were qualified by a significant interaction between externalizing symptoms and ADHD medication (B = −0.250; SE = 0.086; *P* = .004) (Table 2 and the Figure), such that for children who were not receiving ADHD medications, more externalizing symptoms were associated with greater odds of suicidality (for a change of 1 SD in externalizing symptoms, OR, 1.42; 95% CI, 1.33-1.52; *P* < .001). However, for children who were receiving ADHD medication, there was no association (OR, 1.15; 95% CI, 0.97-1.35; *P* = .10).

**Table 2.** Association of ADHD Medication Use With Externalizing Symptoms and Suicidality Reported at Baseline Adolescent Brain Cognitive Development Study Assessment

Variable	Adjusted model 1^**a**^					Adjusted model 2^**b**^				
	B	SE	Wald	OR (95% CI)	*P* value	B	SE	Wald	OR (95% CI)	*P* value
Externalizing symptoms^c^	0.29	0.03	97.51	1.34 (1.26-1.42)	<.001	0.21	0.03	45.94	1.23 (1.16-1.31)	<.001
Any ADHD medication	0.27	0.11	5.97	1.32 (1.06-1.64)	.01	0.13	0.12	1.33	1.14 (0.91-1.43)	.25
Externalizing by ADHD medications^d^	−0.25	0.09	8.36	0.78 (0.66-0.92)	.004	−0.18	0.09	4.22	0.83 (0.7-0.99)	.04
Female sex	−0.11	0.07	2.64	0.89 (0.78-1.02)	.10	0.08	0.07	1.13	1.08 (0.94-1.24)	.29
Black race	0	0.12	0	1 (NA)	.99	−0.11	0.12	0.89	0.89 (0.71-1.13)	.35
Asian race	0.16	0.14	1.32	1.17 (0.89-1.54)	.25	0.15	0.14	1.06	1.16 (0.88-1.53)	.30
Hispanic ethnicity	−0.02	0.09	0.04	0.98 (0.82-1.18)	.84	0.02	0.10	0.05	1.02 (0.85-1.23)	.83
Parents divorced or separated	0.12	0.12	1.00	1.13 (0.89-1.42)	.32	0.10	0.12	0.69	1.11 (0.87-1.40)	.41
Receiving antidepressant or antipsychotic medication	1.05	0.16	43.37	2.85 (2.09-3.89)	<.001	1.10	0.16	44.98	2.99 (2.17-4.12)	<.001
Family conflict	NA	NA	NA	NA	NA	0.17	0.02	104.30	1.18 (1.15-1.22)	<.001
Weekend screen use	NA	NA	NA	NA	NA	0.04	0.01	22.75	1.04 (1.03-1.06)	<.001
Parental supervision	NA	NA	NA	NA	NA	−0.36	0.06	31.44	0.70 (0.62-0.79)	<.001
Positive school involvement	NA	NA	NA	NA	NA	−0.09	0.01	45.82	0.91 (0.89-0.94)	<.001

Abbreviations: ADHD, attention-deficit/hyperactivity disorder; NA, not applicable; OR, odds ratio; SE, standard error.

^a^ Model 1 included age, parents’ education, parents’ marital status (married vs not), and race (White, Black, Asian, or other [ie, American Indian, Native Hawaiian, and a category reported by participants as *other*]) and Hispanic ethnicity.

^b^ Model 2 is similar to model 1 in addition to the 4 risk and protective factors described previously^34^: family conflict, weekend screen time, parental supervision, and positive school involvement.

^c^ To improve interpretability of externalizing symptoms main association, ADHD medication variable was regressed out of the sum of externalizing symptoms (resulting in a *z* score), such that the obtained OR reflect a change in odds for a change in 1 SD of externalizing symptoms.

^d^ Interaction term was introduced in a separate model.

**Figure.**
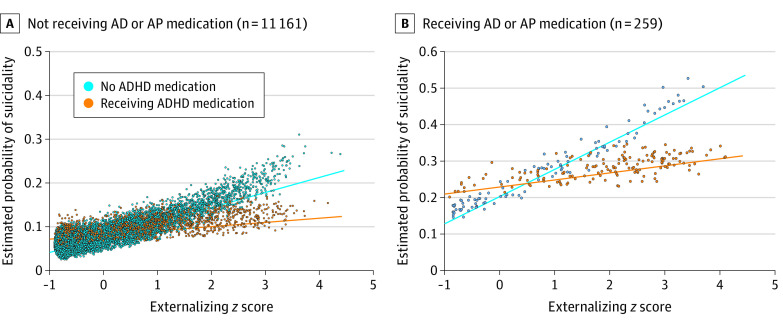
Association of Attention-Deficit/Hyperactivity Disorder (ADHD) Medications With Externalizing Symptoms and Baseline Suicidality in Adolescent Brain Cognitive Development Study Participants Scatter plots and linear regression lines show estimated probabilities of baseline suicidality in 11 161 children not receiving antidepressant (AD) or antipsychotic (AP) medication (A) and 259 children receiving AD or AP medication (B). Externalizing symptoms included ADHD hyperactivity symptoms, oppositional defiant disorder symptoms, and conduct disorder symptoms.

The association of ADHD medication was similar in both the population of 11 161 children not receiving additional psychiatric medication (Figure, panel A) and the 259 children receiving AD or AP medication (Figure, panel B, and eTable 5 in the Supplement) (nonsignificant symptoms by ADHD medication by AD or AP medication interaction, OR, 0.892; 95% CI, 0.550-1.335; *P* = .64), and was observed similarly across sexes (eTable 6 in the Supplement) (nonsignificant symptoms by ADHD medication by sex interaction, OR, 1.041; 95% CI, 0.696-1.557; *P* = .84).

### Association of Externalizing Symptoms and ADHD Medications With Suicidality at 1-Year Follow-up Assessment

We then tested whether the ADHD medication treatment at baseline would moderate the association of externalizing symptoms (at baseline) with suicidality reported at 1-year follow-up assessment. Longitudinal 1-year follow-up data were available for 11 014 participants, who had similar baseline ADHD medication use and suicidality and slightly fewer externalizing symptoms compared with the 790 participants (7.1%) who were lost to follow-up (eTable 7 in the Supplement). In line with our preregistered hypothesis, children with high externalizing symptom loads who were treated with ADHD medications at baseline were less likely to report current suicidality at longitudinal assessment (ie, significant symptom-by-medication interaction; B = −0.34; SE = 0.18; 1-tailed test *P* = .03) (Table 3 and eFigure in the Supplement), with the model covarying for baseline suicidality and all confounders as in models described previously. For children who were not receiving ADHD medications at baseline, there was an association between higher externalizing symptoms and greater odds of current suicidality at the 1-year follow-up (OR, 1.33; 95% CI, 1.14-1.55; *P* < .001). However, for children who were receiving ADHD medication, there was no such association (OR, 0.89; 95% CI, 0.63-1.25; *P* = .50).

**Table 3.** Association of Baseline ADHD Medication Use With Baseline Externalizing Symptoms and Report of Current Suicidality at 1-Year Follow-up

Variable	Model 1^**a**^					Model 2^**b**^				
	B	SE	Wald	OR (95%CI)	*P* value^**c**^	B	SE	Wald	OR (95%CI)	*P* value^**c**^
Externalizing symptoms^d^	0.2	0.07	8.77	1.22 (1.07-1.39)	.003	0.12	0.07	3.13	1.13 (0.99-1.29)	.08
Any ADHD medication	0.61	0.23	7.18	1.84 (1.18-2.88)	.007	0.44	0.23	3.59	1.56 (0.99-2.45)	.06
Externalizing by ADHD medications^e^	−0.34	0.18	3.53	0.71 (0.50-1.01)	.03	−0.29	0.19	2.48	0.75 (0.52-1.07)	.06

Abbreviations: ADHD, attention-deficit/hyperactivity disorder; OR, odds ratio; SE, standard error.

^a^ Binary logistic regression model with current suicidality at 1-year follow-up assessment as the dependent variable, testing main association and interaction of baseline externalizing symptom count and ADHD medication (binary variable). Model covaried for age, parents’ education, marital status, race (White, Black, Asian, or other [ie, American Indian, Native Hawaiian, and a category reported by participants as *other*]), Hispanic ethnicity, time between baseline and follow-up assessment, and suicidality at baseline assessment.

^b^ Model 2 is similar to 1 in addition to the 4 risk and protective factors described previously in ABCD: family conflict, weekend screen time, parental supervision, and positive school involvement.^34^

^c^ One-tailed test for the interaction association based on anticipated direction of medication protective association, as observed in cross-sectional model findings, and based on a preregistered hypothesis for protective association of ADHD medication in children with high externalizing symptoms.

^d^ To improve interpretability of externalizing symptoms main association, ADHD medication variable was regressed out of the sum of externalizing symptoms (resulting in a *z* score), hence that the obtained OR reflect a change in odds for a change in 1 SD of externalizing symptoms.

^e^ Interaction term was introduced in a separate model.

### Sensitivity Analyses

#### Association of Established Risk and Protective Factors With Suicidality in the ABCD Study

Results from the main analyses were significant when accounting for potential confounding associations of family dynamics and school factors in the cross-sectional model (ie, symptom-by-medication interaction, B = −0.18; SE = 0.09; *P* = .04) (Table 2). However, there was no association with 1-year follow-up suicidality (B = −0.29; SE = 0.19; 1-tailed test, *P* = .06) (Table 3).

#### Matched Analysis

Because children receiving ADHD medication differed from those not receiving ADHD medication (eTable 8 in the Supplement), we matched participants at different thresholds of externalizing symptoms (>1 SD, >2 SD, and >3 SD), where we expected to detect associations of ADHD medication with suicidality according to the adjusted model visualized in the Figure. Results revealed that the lower rates of suicidality in children receiving ADHD medication, compared with nontreated children, was most pronounced among children with the greatest symptom load, in both the cross-sectional and the 1-year follow-up assessment (Table 4). Specifically, in children with more than 3 SD externalizing symptom loads not receiving AD or AP medications, there was 1 child who reported suicidality in the group of 35 participants receiving ADHD medications, compared with 6 participants reporting suicidality among the 35 matched controls not receiving ADHD medication (relative risk, 0.17; 95% CI, 0.02-1.31; 1-sided *P* = .04).

**Table 4.** Matched Comparison of Children With High Externalizing Symptoms Receiving ADHD Medications Compared With Controls

Comparison	Participants, No. (%)							
	Not receiving AD or AP medications (n = 11 590)				Receiving AD or AP medications (n = 271)			
	Not receiving ADHD medications^**a**^	Receiving ADHD medications^**b**^	RR (95% CI)	*P* value^**c**^	Not receiving ADHD medications^**a**^	Receiving ADHD medications^**b**^	RR (95% CI)	*P* value^**c**^
>1 SD and above (≥10 symptoms) (^d^	391 (100.00)	391 (100.00)	NA	NA	40 (100.00)	40 (100.00)	NA	NA
Cases of baseline suicidality^e^	65 (16.60)	58 (14.80)	0.89 (0.64-1.24)	.25	15 (38.00)	13 (32.50)	0.87 (0.48-1.58)	.32
Cases of 1-y suicidality^e^	66 (16.90)	51 (13.00)	0.77 (0.55-1.08)	.07	12 (30.00)	10 (25.00)	0.83 (0.41-1.70)	.31
>2 SD and above (≥16 symptoms)^d^	221 (100.00)	221 (100.00)	NA	NA	23 (100.00)	23 (100.00)	NA	NA
Cases of baseline suicidality^e^	44 (19.90)	32 (14.50)	0.73 (0.48-1.02)	.07	8 (34.80)	4 (17.40)	0.5 (0.17-1.43)	.10
Cases of 1-y suicidality^e^	41 (18.60)	25 (11.30)	0.61 (0.38-0.97)	.02	6 (21.60)	4 (17.40)	0.67 (0.22-2.05)	.24
>3 SD and above (≥22 symptoms)^d^	35 (100.00)	35 (100.00)	NA	NA	NA^f^	NA^f^	NA^f^	NA^f^
Cases of baseline suicidality^e^	6 (17.10)	1 (2.90)	0.17 (0.02-1.31)	.04	NA^f^	NA^f^	NA^f^	NA^f^
Cases of 1-y suicidality^e^	5 (14.30)	1 (2.90)	0.2 (0.02-1.63)	.07	NA^f^	NA^f^	NA^f^	NA^f^

Abbreviations: AD, antidepressants; ADHD, attention-deficit/hyperactivity disorder; AP, antipsychotics; RR, relative risk.

^a^ Participants receiving ADHD medications were matched to children not receiving ADHD on multiple parameters, including age, sex, race, ethnicity, parents’ education and marital status, family conflict, parental supervision, weekend screen time, and positive school involvement.

^b^ ADHD medications included methylphenidate and amphetamine derivatives, α-2 agonists, and atomoxetine.

^c^ Uncorrected 1-sided test confirming preregistered hypothesis and adjusted regression models.

^d^ Symptoms included ADHD hyperactivity symptoms, oppositional defiant disorder symptoms, and conduct disorder symptoms, as in adjusted regression models.

^e^ Suicidality cases were defined as endorsement of past or current suicidal ideation or attempt.

^f^ Total participants in this group included 32 participants of whom only 7 participants did not receive ADHD medication; therefore, no statistical test was conducted on this group.

#### Additional Sensitivity Analyses

We ran several variations of the main model (1) controlling for baseline history of depression or anxiety (eTable 9 in the Supplement); (2) using different combinations of externalizing symptoms, including inattention symptoms or excluding ODD and CD symptoms (eTable 10 in the Supplement); and (3) using youth-reported suicidal ideation (eTable 11 in the Supplement) or (4) parent-reported suicidality (eTable 12 in the Supplement) instead of youth-reported suicidality as in the main analysis. We also ran all main analyses using an imputed data set (eTable 13 in the Supplement) and a data set that excluded family-related children or included 1 randomly selected child from a family (eTable 14 in the Supplement). All models revealed findings similar to those of the main analyses.

### Exploratory Evaluation of Medication Class’s Association

We tested the associations of methylphenidate medications (541 participants), amphetamine salts (363 participants), α-2-agonists (243 participants), and atomoxetine (47 participants) in separate models. All classes showed negative B values for the interaction term of externalizing symptoms and medication, but only methylphenidate showed a statistically significant interaction (B = −0.416; SE = 0.112; *P* < .001) (eTable 15 in the Supplement).

## Discussion

Leveraging the ABCD Study cohort, we document that ADHD medication is associated with less suicidality in preadolescent children with externalizing symptoms (measured dimensionally combining hyperactivity ADHD symptoms and ODD and CD symptoms). The observed difference in suicidality between children receiving and not receiving ADHD medication was greatest among children with the most externalizing symptoms. Findings were replicated in a 1-year longitudinal analysis and were robust to multiple confounders. As the reported prevalence of ADHD medication use and suicidality are consistent with recent literature,^32,38^ the ABCD Study sample appears to be representative for the purpose of the current analyses.

Our findings address a knowledge gap on childhood suicidality that is critical for several reasons.^8^ First, childhood suicidality has rapidly increased over the last decade.^2,3,4,5,6,7^ Second, the results offer a straightforward, actionable target to optimize prevention plans and intervention strategies in elementary school–age children with externalizing problems. Early diagnosis and treatment with ADHD medication, particularly among children with severe externalizing symptoms, may serve not only to improve learning and behavior problems,^39,40^ but also to decrease suicidality risk. Third, childhood suicidality is associated with adult psychiatric morbidity and mortality and, thus, may represent an early marker for lifelong vulnerability to poor mental health.^41,42^ Thus, better and more thorough screening of school-age children for externalizing psychopathology, which is a modifiable developmental risk factor, has strong potential to prevent and mitigate serious psychopathology later in the life span.

This study supports a dimensional approach to ADHD.^43^ Observing externalizing disorders along a continuum holds promise for elucidating more precise associations with other forms of psychopathology, including suicidality. Notably, interpretation of the dimensional approach in the context of medication treatment should be done cautiously, because, to our knowledge, there are no clinical guidelines for treatment of externalizing symptoms alone with pharmacotherapy, but rather, these medications are used to treat children with an ADHD diagnosis. The fact that the associations were similar among boys and girls is also noteworthy, especially in light of data showing that girls are less likely to receive ADHD treatment compared with boys.^44^

An exploratory analysis of the associations of different classes of ADHD medication with suicidality found a significant association with methylphenidate, but not for atomoxetine, amphetamine salts, or α-2-agonists. Given the limited sample sizes of these other drug classes, it is possible that lack of statistical power underlies these findings, but smaller or different associations with these medications cannot be ruled out. In addition, amphetamines and α-2-agonists may be prescribed differently than methylphenidate (eg, as second-line treatments),^45^ confounding their associations by indication. Our findings for methylphenidate are in line with prior evidence supporting the effectiveness of methylphenidate in reducing impulsivity,^46^ which contributes to the suicide risk–bearing association of ADHD in youth.^47,48,49^ Further studies are needed to better understand the mechanisms underlying the potential protective association of methylphenidate vs other medications with youth suicidality.

This study has immediate clinical implications that should be viewed in the context of the observational nature of the study, which was not designed to test causal effects of treatment. Although the reference standard for establishing protective effects of medications are randomized, placebo-controlled trials, it is unlikely that psychotropic intervention trials for suicidality can be used in the general pediatric population, as rates of childhood suicidality are low (approximately 8% lifetime and <1.5% current in the ABCD Study). A major challenge is to draw causal inferences from observational data to better inform clinical decision-making,^50^ thereby complementing the evidence derived from randomized, placebo-controlled trials, which themselves are not free from limitations.^51^ Thus, by leveraging the ABCD Study cohort, we bridge a critical knowledge gap regarding modifiable pediatric suicide risk factors in children. We suggest that the replication of our main finding in the longitudinal 1-year assessment and the matched sensitivity analyses support the applicability of findings to real-life clinical practice.

### Limitations

Our findings should be interpreted considering several limitations. First, we cannot rule out the role of confounders (measured and unmeasured) that are associated with ADHD medication treatment and suicidality, such as other modalities of mental health interventions, which may have affected the results. Specifically, it is possible that higher-risk children were more likely to be prescribed medications, creating a confounding-by-indication problem. However, we included other psychiatric medication treatment (AD or AP) and multiple established risk and protective factors for suicidality that may somewhat mitigate this concern. Second, the association of medication in the longitudinal and matched analyses was tested using a 1-tailed test (in line with our preregistered hypothesis). Third, the participants lost to follow-up (7.1%) reported slightly more externalizing symptoms, which might have affected the longitudinal findings. Fourth, longitudinal models did not reflect the outcomes of ongoing ADHD medication treatment or of changes in externalizing symptoms over time, as both medication use and externalizing symptoms were assessed at baseline. Future analyses of the ABCD Study cohort and other longitudinal data sets are warranted to examine suicidality trajectories throughout adolescence, which heralds higher rates of suicidality^4^ and more power to study modifiable risk factors. Despite these limitations, the size, representativeness, and systematic in-depth assessment of the ABCD Study cohort are unique strengths, allowing us to quantify the associations of ADHD pharmacotherapy with child suicidality, over and above the outcomes of established risk and protective factors and other potential confounders.

## Conclusions

In conclusion, this study found that ADHD medication was associated with less suicidality in children with high externalizing symptom burden, equally for both sexes. The findings reported here may provide immediate and practical implications to potentially reduce childhood suicidality. Given the high prevalence of externalizing disorders among children, including ADHD, ODD, and CD, it is critical that we optimize both psychoeducation and pharmacotherapy interventions to shift otherwise adverse developmental trajectories and improve functional and clinical outcomes.

## Notes

Supplement. eMethods. eReferences. **eTable 1.** Symptoms Included in the Externalizing Symptoms Variable **eTable 2.** Definitions of Medication Variables **eTable 3.** Suicidal Ideation and Suicide Attempts Reported by ABCD Participants **eTable 4.** Diagnoses Used to Derive Depression and Anxiety Variables **eTable 5.** Antidepressant- and Antipsychotic- Treated Children: Stratified Analysis and 3-Way Interaction Model **eTable 6.** Sex-Stratified Analysis and 3-Way Interaction Model **eTable 7.** Demographic and Clinical Comparison Between Participants With Baseline and 1-Year Follow-up Data and Participants With Only Baseline Data (Lost to Follow-up) **eTable 8. **Demographic and Clinical Comparisons of Children With and Without ADHD Medication in ABCD and Following Matching Based on Externalizing Symptom Load **eTable 9.** Effect of Baseline Depression and Anxiety on Main Model Results **eTable 10.** Effect of Different Definitions of Externalizing Symptoms on Main Model Results **eTable 11.** Sensitivity Analysis Using Suicidal Ideation (SI) as the Dependent Variable Instead of Suicidality (i.e., SI or Suicide Attempt) as in Main Model **eTable 12. **Sensitivity Analysis Using Parent-Report Suicidality as the Dependent Variable Instead of Child-Report Suicidality as in Main Model **eTable 13.** Imputed Data Sensitivity Analysis **eTable 14.** Family Relatedness Sensitivity Analysis **eTable 15.** Exploratory Analyses of Different ADHD Medication Class **eFigure.** Moderating Effect of ADHD Medication Use at Baseline on the Relationship Between Baseline Externalizing Symptoms and Suicidality in 1-Year Follow-up Assessment
