# Supplementary material for: Evaluation of Attention-Deficit/Hyperactivity Disorder Medications, Externalizing Symptoms, and Suicidality in Children
Source: JAMA Netw Open. 2021 Jun 4;4(6):e2111342. doi: 10.1001/jamanetworkopen.2021.11342 (PMC8178707; doi:10.1001/jamanetworkopen.2021.11342)
Supplement: Supplement. — eMethods. eReferences. eTable 1. Symptoms Included in the Externalizing Symptoms Variable eTable 2. Definitions of Medication Variables eTable 3. Suicidal Ideation and Suicide Attempts Reported by ABCD Participants eTable 4. Diagnoses Used to Derive Depression and Anxiety Variables eTable 5. Antidepressant- and Antipsychotic- Treated Children: Stratified Analysis and 3-Way Interaction Model eTable 6. Sex-Stratified Analysis and 3-Way Interaction Model eTable 7. Demographic and Clinical Comparison Between Participants With Baseline and 1-Year Follow-up Data and Participants With Only Baseline Data (Lost to Follow-up) eTable 8. Demographic and Clinical Comparisons of Children With and Without ADHD Medication in ABCD and Following Matching Based on Externalizing Symptom Load eTable 9. Effect of Baseline Depression and Anxiety on Main Model Results eTable 10. Effect of Different Definitions of Externalizing Symptoms on Main Model Results eTable 11. Sensitivity Analysis Using Suicidal Ideation (SI) as the Dependent Variable Instead of Suicidality (i.e., SI or Suicide Attempt) as in Main Model eTable 12. Sensitivity Analysis Using Parent-Report Suicidality as the Dependent Variable Instead of Child-Report Suicidality as in Main Model eTable 13. Imputed Data Sensitivity Analysis eTable 14. Family Relatedness Sensitivity Analysis eTable 15. Exploratory Analyses of Different ADHD Medication Class eFigure. Moderating Effect of ADHD Medication Use at Baseline on the Relationship Between Baseline Externalizing Symptoms and Suicidality in 1-Year Follow-up Assessment [file jamanetwopen-e2111342-s001.pdf]

Supplemental Online Content

Shoval G, Visoki E, Moore TM, et al. Evaluation of attention-deficit/hyperactivity disorder medications, externalizing symptoms, and suicidality in children. *JAMA Netw Open*. 2021;4(6):e2111342. doi:10.1001/jamanetworkopen.2021.11342

eMethods.

eReferences.

eTable 1. Symptoms Included in the Externalizing Symptoms Variable

eTable 2. Definitions of Medication Variables

eTable 3. Suicidal Ideation and Suicide Attempts Reported by ABCD Participants

eTable 4. Diagnoses Used to Derive Depression and Anxiety Variables

eTable 5. Antidepressant- and Antipsychotic- Treated Children: Stratified Analysis and 3-Way Interaction Model

eTable 6. Sex-Stratified Analysis and 3-Way Interaction Model

eTable 7. Demographic and Clinical Comparison Between Participants With Baseline and 1-Year Follow-up Data and Participants With Only Baseline Data (Lost to Follow-up)

eTable 8. Demographic and Clinical Comparisons of Children With and Without ADHD Medication in ABCD and Following Matching Based on Externalizing Symptom Load

eTable 9. Effect of Baseline Depression and Anxiety on Main Model Results

eTable 10. Effect of Different Definitions of Externalizing Symptoms on Main Model Results

eTable 11. Sensitivity Analysis Using Suicidal Ideation (SI) as the Dependent Variable Instead of Suicidality (i.e., SI or Suicide Attempt) as in Main Model

eTable 12. Sensitivity Analysis Using Parent-Report Suicidality as the Dependent Variable Instead of Child-Report Suicidality as in Main Model

eTable 13. Imputed Data Sensitivity Analysis

eTable 14. Family Relatedness Sensitivity Analysis

eTable 15. Exploratory Analyses of Different ADHD Medication Class

eFigure. Moderating Effect of ADHD Medication Use at Baseline on the Relationship Between Baseline Externalizing Symptoms and Suicidality in 1-Year Follow-up Assessment

This supplemental material has been provided by the authors to give readers additional information about their work.

© 2021 Shoval G et al. *JAMA Network Open.*

eMethods.

Sensitivity analyses

We conducted several sensitivity analyses for the main model. To evaluate robustness of results, we included established risk and protective factors for suicidality previously described in ABCD.[1] To probe the effects of our externalizing symptoms exposure variable, given established heterogeneity in the clinical phenotype,[2] we ran the main model using combinations of different symptom dimensions (ADHD including/excluding inattention, ODD and CD). To address effect of our choice of the dependent variable (child report suicidality), we ran two models using two different suicide measures: child report suicidal ideation and parent report suicidality. To control for depression/anxiety, we included in the main model KSADS-based depression/anxiety diagnoses. As an alternative to our handling of missing data using listwise deletion in the main analyses, we conducted sensitivity analyses running all models using an imputed dataset (created using the R library Amelia). To account for potential family-relatedness effects, we analyzed data including only one participant from a family, or excluding any related children, similar to previous work on ABCD suicidality.[1]

Lastly, in attempt to better estimate clinical presentations where ADHD medication is especially associated with less suicidality, we matched participants receiving ADHD medications with high symptom loads at 3 thresholds (>1/2/3 SDs) with untreated controls and compared suicidality rates.

Exploratory analyses

To evaluate the moderating effect of sex on the main study question (interaction effect of symptoms and ADHD medication), we ran the main model adding the 3-way

© 2021 Shoval G et al. *JAMA Network Open.*

interaction term of externalizing symptoms x ADHD medications x sex (including all of the 2-way interactions: symptoms x ADHD medications; externalizing x sex; ADHD medications x sex). A significant 3-way interaction would indicate that the moderating effect of ADHD medication on the relationship between externalizing symptoms and suicidality is different between the sexes. In addition, we ran sex-stratified analyses of the main model. This approach was also employed to examine effects in the children receiving any AD/AP medication.

To explore potential differences in the moderating effects of different ADHD medication classes on the association between externalizing symptoms and suicidality, we also ran the main analysis as four separate models (i.e., one for each medication class).

eREFERENCES

1. Janiri D, Doucet GE, Pompili M, et al. Risk and protective factors for childhood suicidality: a US population-based study. *Lancet Psychiatry*. 2020;7(4):317-326. doi:10.1016/S2215-0366(20)30049-3
2. Wilson S, Hopwood CJ, McGue M, Iacono WG. Personality Heterogeneity in Adolescents With Disruptive Behavior Disorders1. *J Res Personal*. 2019;82. doi:10.1016/j.jrp.2019.103851

© 2021 Shoval G et al. *JAMA Network Open.*

eTable 1. Symptoms Included in the Externalizing Symptoms Variable

Symptom	Disorder	ABCD Identifier	Temporal Identifier
Acts like driven by a motor	ADHD	ksads_14_403_p	Present
	ADHD	ksads_14_419_p	Past
Blurts out answers	ADHD	ksads_14_405_p	Present
	ADHD	ksads_14_421_p	Past
Difficulty waiting turn	ADHD	ksads_14_406_p	Present
	ADHD	ksads_14_422_p	Past
Difficulty playing quietly	ADHD	ksads_14_404_p	Present
	ADHD	ksads_14_420_p	Past
Difficulty remaining seated	ADHD	ksads_14_84_p	Present
	ADHD	ksads_14_86_p	Past
	ADHD	ksads_14_85_p	Present (since elementary school)
	ADHD	ksads_14_87_p	Past (>1 school year)
Fidgets	ADHD	ksads_14_401_p	Present
	ADHD	ksads_14_417_p	Past
Impulsivity	ADHD	ksads_14_88_p	Present
	ADHD	ksads_14_89_p	Past
	ADHD	ksads_14_90_p	Past (>1 school year)
Often talks excessively	ADHD	ksads_14_408_p	Present
	ADHD	ksads_14_424_p	Past
Often interrupts or intrudes on others	ADHD	ksads_14_407_p	Present
	ADHD	ksads_14_423_p	Past
Runs or climbs excessively	ADHD	ksads_14_402_p	Present
	ADHD	ksads_14_418_p	Past
Often disobeys rules/requests	ODD	ksads_15_95_p	Present
	ODD	ksads_15_96_p	Past

© 2021 Shoval G et al. *JAMA Network Open.*

eTable 1. (cont.)

Symptom	Disorder	ABCD Identifier	Temporal Identifier
Often blames others for own mistakes	ODD	ksads_15_436_p	Present
	ODD	ksads_15_443_p	Past
Often deliberatively annoys people	ODD	ksads_15_435_p	Present
	ODD	ksads_15_442_p	Past
Often angry or resentful	ODD	ksads_15_433_p	Present
	ODD	ksads_15_440_p	Past
Often argues with adults/authority	ODD	ksads_15_93_p	Present
	ODD	ksads_15_94_p	Past
Often touchy or easily annoyed	ODD	ksads_15_432_p	Present
	ODD	ksads_15_439_p	Past
Often loses temper	ODD	ksads_15_91_p	Present
	ODD	ksads_15_92_p	Past
Causes problems in functioning or distress in self or others	ODD	ksads_15_437_p	Present
	ODD	ksads_15_444_p	Past
Spiteful or vindictive	ODD	ksads_15_434_p	Present
	ODD	ksads_15_441_p	Past
Breaking and entering	CD	ksads_16_449_p	Present
	CD	ksads_16_450_p	Past
Forced someone into sexual activity	CD	ksads_16_463_p	Present
	CD	ksads_16_464_p	Past
Fire setting	CD	ksads_16_453_p	Present
	CD	ksads_16_454_p	Past
Has been physically cruel to people	CD	ksads_16_461_p	Present
	CD	ksads_16_462_p	Past
Has been physically cruel to animals	CD	ksads_16_465_p	Present
	CD	ksads_16_466_p	Past

© 2021 Shoval G et al. *JAMA Network Open.*

eTable 1. (cont.)

Symptom	Disorder	ABCD Identifier	Temporal Identifier
Often Lies	CD	ksads_16_98_p	Present
	CD	ksads_16_99_p	Past
Often bullies others	CD	ksads_16_104_p	Present
	CD	ksads_16_105_p	Past
Often initiates physical fights	CD	ksads_16_102_p	Present
	CD	ksads_16_103_p	Past
Ran away overnight	CD	ksads_16_457_p	Present
	CD	ksads_16_458_p	Past
Stays out late at night despite parental prohibition	CD	ksads_16_455_p	Present
	CD	ksads_16_456_p	Past
Stealing while confronting victim	CD	ksads_16_451_p	Present
	CD	ksads_16_452_p	Past
Stealing	CD	ksads_16_106_p	Present
	CD	ksads_16_107_p	Past
Truancy	CD	ksads_16_100_p	Present
	CD	ksads_16_101_p	Past
Vandalism	CD	ksads_16_447_p	Present
	CD	ksads_16_448_p	Past
Use of a weapon that can cause serious harm	CD	ksads_16_459_p	Present
	CD	ksads_16_460_p	Past

The externalizing symptoms variable was defined as the sum of externalizing hyperactivity symptoms from ADHD, and all symptoms of ODD and conduct disorder items in the K-SADS. Present and past were combined as one symptom; combination of 0 and NA was defined as 0. The variable was scored on a discrete scale from 0 - 34 based on presence of symptoms in the table.
Abbreviations: ADHD = Attention Deficit Hyperactivity Disorder; ODD = Oppositional Defiant Disorder; CD = Conduct Disorder

© 2021 Shoval G et al. *JAMA Network Open.*

eTable 2. Definitions of Medication Variables

	Medication	Active Compound(s)	Drug Class
ADHD medication	Ritalin	Methylphenidate HCl	Methylphenidate Derivative (MPH)
	Quillivant		
	Quillichew		
	Methylin		
	Concerta		
	Metadate		
	Aptensio		
	Daytrana		
	Focalin	Dexmethylphenidate HCl	
	Adderall	Dextroamphetamine Saccharate, Amphetamine Aspartate, Dextroamphetamine Sulfate, Amphetamine Sulfate	Amphetamine (AMPH)
	Adzenys		
	Dynavel		
	Evekeo		
	Zenzedi	Dextroamphetamine Sulfate	
	Dexedrine		
	Procentra		
	Vyvanse	Lisdexamfetamine Dimesylate	
	Intuniv	Guanfacine	Alpha Agonist
	Tenex	Guanfacine HCl	
	Catapres	Clonidine HCl	
	Nexiclon		
	Strattera	Atomoxetine HCl	Atomoxetine

© 2021 Shoval G et al. *JAMA Network Open.*

eTable 2. (cont.)

	Medication	Active Compound(s)	Drug Class
**Antipsychotic medication**	Abilify	Aripiprazole	Antipsychotic
	Risperdal	Risperidone	
	Latuda	Lurasidone HCl	
	Ziprasidone	Ziprasidone HCl	
	Clozapine	Clozapine	
	Seroquel	Quetiapine	
**Antidepressant medication**	Prozac	Fluoxetine HCl	SSRI
	Sarafem		
	Zoloft	Sertraline HCl	
	Fluvoxamine	Fluvoxamine Maleate	
	Celexa	Citalopram HBr	
	Lexapro	Escitalopram Oxalate	
	Amitriptyline	Amitriptyline HCl	Other Antidepressant
	Imipramine	Imipramine HCl	
	Venlafaxine	Venlafaxine HCl	
	Desvenlafaxine	Desvenlafaxine Succinate	
	Remeron	Mirtazapine	
	Trazodone	Trazodone HCl	
	Buspar	Buspirone	
	Wellbutrin	Bupropion HCl	
	Buproban	Bupropion HBr	

The ADHD medication use variable (binary yes/no) was based on the child receiving any medication of the four defined ADHD medication classes.
The antidepressant/antipsychotic variable (binary yes/no) was based on the child receiving any antidepressant or antipsychotic medication.
Abbreviations: ADHD = Attention Deficit Hyperactivity Disorder; SSRI = Selective Serotonin Reuptake Inhibitor

© 2021 Shoval G et al. *JAMA Network Open.*

eTable 3. Suicidal Ideation and Suicide Attempts Reported by ABCD Participants

		n	% of sample
**Baseline assessment**	Ideation past	903	7.60%
	Ideation current	264	2.22%
	Attempt past	129	1.08%
	Attempt current	47	0.40%
**1-year follow-up assessment**	Ideation past	840	7.58%
	Ideation current	193	1.74%
	Attempt past	135	1.22%
	Attempt current	29	0.26%

Prevalence of suicidal ideation and attempts are shown at baseline assessment and at 1-year follow-up for the total sample.
Total baseline sample included N=11 878. Missing data for suicidality measures was 0.6% at baseline. Longitudinal data on suicidality was available for n= 11 077 (missing for 6.7% of the original cohort).
Current refers to past 2 weeks.

© 2021 Shoval G et al. *JAMA Network Open.*

eTable 4. Diagnoses Used to Derive Depression and Anxiety Variables

Binary variable (yes/no)	ABCD KSADS derived diagnosis	ABCD element name
Depression	Major Depressive Disorder Present	ksads_1_840_t
	Major Depressive Disorder, Current, in Partial Remission (F32.4)	ksads_1_841_t
	Major Depressive Disorder, Past (F32.9)	ksads_1_842_t
	Persistent Depressive Disorder (Dysthymia) Present F34.1	ksads_1_843_t
	Persistent Depressive Disorder (Dysthymia) In Partial Remission F34.1	ksads_1_844_t
	Persistent Depressive Disorder (Dysthymia) Past F34.1	ksads_1_845_t
	Unspecified Depressive Disorder Current (F32.9)	ksads_1_846_t
	Unspecified Depressive Disorder Past (F32.9)	ksads_1_847_t
Anxiety	Social Anxiety Disorder (F40.10) Present	ksads_8_863_t
	Social Anxiety Disorder (F40.10) Past	ksads_8_864_t
	Generalized Anxiety Disorder Present (F41.1)	ksads_10_869_t
	Generalized Anxiety Disorder Past (F41.1)	ksads_10_870_t
	Other Specified Anxiety Disorder (Social Anxiety Disorder, impairment, does not meet minimum duration) F41.8	ksads_8_911_t
	Other Specified Anxiety Disorder (Social Anxiety Disorder, impairment, does not meet minimum duration), Past, F41.8	ksads_8_912_t
	Other Specified Anxiety Disorder (Generalized Anxiety Disorder, impairment, does not meet minimum duration) F41.8	ksads_10_913_t
	Other Specified Anxiety Disorder (Generalized Anxiety Disorder, impairment, does not meet minimum duration), Past, F41.8	ksads_10_914_t

We considered positive history of depression for every participant who was given any of the above diagnoses (coded as 1 in any of the ABCD elements above). History of anxiety was determined similarly using the above anxiety diagnoses.

© 2021 Shoval G et al. *JAMA Network Open.*

eTable 5. Antidepressant- and Antipsychotic- Treated Children: Stratified Analysis and 3-Way Interaction Model

**Interaction model**[a]	**Total sample (N=11 420)**						
	B	SE	Wald	OR	LL 95% CI	UL 95% CI	*p*-value
Externalizing symptoms[b]	0.293	0.030	97.946	1.340	1.265	1.420	<.001
Any ADHD medication	0.293	0.112	6.847	1.340	1.076	1.669	0.009
Receiving AD/AP	1.037	0.159	42.679	2.821	2.067	3.850	<.001
Ext. symp. by ADHD meds by AD/AP meds	-0.115	0.246	0.217	0.892	0.550	1.445	0.642
**Stratified analysis**	**No AD/AP (n=11 161)**						
	B	SE	Wald	OR	LL 95% CI	UL 95% CI	*p*-value
Externalizing symptoms[b]	0.308	0.031	102.041	1.361	1.282	1.445	<.001
Any ADHD medication	0.401	0.117	11.649	1.493	1.186	1.880	0.001
Ext. symp. by ADHD meds	-0.179	0.101	3.165	0.836	0.686	1.018	0.075
**Stratified analysis**	**Receiving AD/AP (n=259)**						
	B	SE	Wald	OR	LL 95% CI	UL 95% CI	*p*-value
Externalizing symptoms[b]	0.070	0.112	0.398	1.073	0.862	1.335	0.528
Any ADHD medication	-0.320	0.315	1.036	0.726	0.392	1.345	0.309
Ext. symp. by ADHD meds	-0.287	0.238	1.451	0.751	0.471	1.197	0.228

[a] Model included age, sex, race, ethnicity, parents' education and marital status. The 3-way interaction was derived from a separate model including main effects and 2-way interactions of all the variables included in the 3-way interaction (i.e., symptoms x ADHD medications; symptoms x AD/AP medications; ADHD medications x AD/AP medications)

[b] To improve interpretability of externalizing symptoms main effect, ADHD medication variable was regressed out of the sum of externalizing symptoms (resulting in a z-score), hence the obtained OR reflect a change in odds for a change in 1SD of externalizing symptoms.

Abbreviations: ADHD = Attention Deficit Hyperactivity Disorder; AD = antidepressants; AP = antipsychotics; ext. symp. = externalizing symptoms; SE= standard error; OR= odds ratio; LL= lower limit; UL= upper limit; CI = confidence interval.
Missing data was 3.9% for the interaction model, 3.7% in stratified model analyzing children without AD/PD and 5.1% in children receiving AD/AP.

© 2021 Shoval G et al. *JAMA Network Open.*

eTable 6. Sex-Stratified Analysis and 3-Way Interaction Model

**Interaction model[a]**	**Total sample (n=11 420)**						
	B	SE	Wald	OR	LL 95% CI	UL 95% CI	*p*-value
Externalizing symptoms[b]	0.293	0.030	97.946	1.340	1.265	1.420	<.001
Any ADHD medication	0.293	0.112	6.847	1.340	1.076	1.669	0.009
Female sex	-0.111	0.069	2.585	0.895	0.782	1.025	0.108
Ext. symp. by ADHD meds by sex	0.040	0.205	0.039	1.041	0.696	1.557	0.844
**Stratified analysis**	**Males (n=5 952)**						
	B	SE	Wald	OR	LL 95% CI	UL 95% CI	*p*-value
Externalizing symptoms[b]	0.227	0.038	36.725	1.255	1.166	1.352	<.001
Any ADHD medication	0.268	0.132	4.136	1.308	1.010	1.694	0.042
Ext. symp. by ADHD meds	-0.261	0.102	6.602	0.770	0.631	0.940	0.010
**Stratified analysis**	**Females (n=5 468)**						
	B	SE	Wald	OR	LL 95% CI	UL 95% CI	*p*-value
Externalizing symptoms[b]	0.405	0.047	72.953	1.500	1.367	1.646	<.001
Any ADHD medication	0.329	0.214	2.358	1.390	0.913	2.116	0.125
Ext. symp. by ADHD meds	-0.186	0.179	1.085	0.830	0.584	1.179	0.298

[a] Model included age, race, ethnicity, parents' education and marital status, and whether child was receiving antidepressants or antipsychotics. The 3-way interactions are derived from a separate model including main effects and 2-way interactions of all the variables included in the 3-way interaction (i.e., symptoms x ADHD medications; symptoms x sex; ADHD medications x sex)
[b] To improve interpretability of externalizing symptoms main effect, ADHD medication variable was regressed out of the sum of externalizing symptoms (resulting in a z-score), hence the obtained OR reflect a change in odds for a change in 1SD of externalizing symptoms.
Abbreviations: ADHD = Attention Deficit Hyperactivity Disorder; AD = antidepressants; AP = antipsychotics; ext. symp. = externalizing symptoms; SE= standard error; OR= odds ratio; LL= lower limit; UL= upper limit; CI = confidence interval. CI = confidence interval.
Missing data was 3.9% for the interaction model, 3.9% in stratified model analyzing boys and 3.8% in model analyzing girls.

© 2021 Shoval G et al. *JAMA Network Open.*

eTable 7. Demographic and Clinical Comparison Between Participants With Baseline and 1-Year Follow-up Data and Participants With Only Baseline Data (Lost to Follow-up)

	Participants with baseline and follow-up data n=11 014	Participants with only baseline data (lost to follow-up) n=790	
	Mean/n (SD/%)	Mean/n (SD/%)	*p*-value[a]
**Age at assessment, years (mean (SD))**	9.91 (0.63)	9.93 (0.61)	0.493
**Sex male, n (%)**	5242 (47.6)	400 (50.6)	0.106
**White, n (%)**	8303 (75.4)	451 (57.1)	<.001
**Black, n (%)**	2221 (20.2)	279 (35.3)	<.001
**Asian, n (%)**	705 (6.4)	40 (5.1)	0.156
**Hispanic, n (%)**	2178 (20.0)	214 (27.6)	<.001
**Parents' education, years (mean (SD))**	16.47 (2.66)	15.18 (2.92)	<.001
**Parents married, n (%)**	7550 (69.0)	396 (51.4)	<.001
**Parents divorced/separated, n (%)**	1404 (12.7)	133 (16.8)	0.001
**Externalizing symptoms (mean (SD))**	4.23 (5.70)	4.86 (6.48)	0.003
**ADHD medications (any), n (%)**	928 (8.4)	64 (8.2)	0.836
**MPH, n (%)**	508 (4.6)	28 (3.6)	0.202
**Amphetamine, n (%)**	322 (2.9)	32 (4.1)	0.086
**Alpha-2-Agonists, n (%)**	220 (2.0)	20 (2.5)	0.357
**Atomoxetine, n (%)**	46 (0.4)	1 (0.1)	0.339
**Receiving other psychiatric meds (any), n (%)**	252 (2.3)	19 (2.4)	0.911
**Antidepressant meds, n (%)**	209 (1.9)	14 (1.8)	0.925
**Antipsychotic meds, n (%)**	64 (0.6)	6 (0.8)	0.687
**Suicidality at baseline, n (%)**	968 (8.8)	72 (9.1)	0.805

[a] T-tests or Chi-square tests, as appropriate.

Participants who were lost to follow up did not differ in terms of age and sex and did not differ in rates of ADHD medication treatment and in suicidality rates. The participants lost to follow up differed in race/ethnicity, socioeconomic status, and had greater externalizing symptomatology.

Abbreviations: ADHD = Attention Deficit Hyperactivity Disorder; MPH = methylphenidate derivative; meds = medications; SD = standard deviation.

© 2021 Shoval G et al. *JAMA Network Open.*

eTable 8. Demographic and Clinical Comparisons of Children With and Without ADHD Medication in ABCD and Following Matching Based on Externalizing Symptom Load

	**Total sample (baseline)**[a]		**No ADHD medication**		**Any ADHD medication**		
	N=11 878		*n=10 856*		*n=1 006*		
	Mean/ n	SD/%	Mean/ n	SD/%	Mean/ n	SD/%	*p*-value[b]
**Age at assessment, years**	9.9	0.6	9.9	0.6	9.9	0.6	0.094
**Sex, female**	5 675	47.8%	5 403	49.7%	272	27.2%	<.001
**White**	8 805	74.1%	8 030	73.9%	767	76.8%	0.048
**Black**	2 518	21.2%	2 262	20.8%	250	25.0%	0.002
**Asian**	745	6.3%	714	6.6%	38	3.8%	0.001
**Hispanic**	2 411	20.3%	2 249	21.0%	159	16.1%	<.001
**Parents' education, years**	16.4	2.7	16.4	2.7	16.2	2.4	0.044
**Parents married**	7 946	67.9%	7 404	68.7%	582	58.7%	<.001
**Parents divorced/separated**	1 537	13.1%	1 363	12.5%	179	17.9%	<.001
**Positive school involvement**	13.3	2.4	13.3	2.3	12.8	2.7	<.001
**Family conflict scale**	2	2	2.0	1.9	2.4	2.0	<.001
**Parental supervision**	4.4	0.5	4.4	0.5	4.2	0.6	<.001
**Weekend screen time**	4.6	3.6	4.5	3.6	5.8	4.2	<.001

Because children that did and did not receive ADHD medication were significantly different in the total sample, we compared subgroups of children with high externalizing factors (>1, 2, and 3 standard deviations from the mean) and use of antidepressant or antipsychotic medications (yes/no). From each of these subgroups, equal amounts of children who did and did not receive ADHD medication were matched based on multiple demographic and clinical variables.

[a] For all variables, missing data was smaller than 1.27% (151 participants out of the 11,878 participants at baseline ABCD assessment).

[b] T-test and Chi-square comparisons for continuous and binary measures, respectively

Abbreviations: ADHD = Attention Deficit Hyperactivity Disorder; AD/AP = antidepressant or antipsychotic medication; SD = standard deviation; NA = not available.

© 2021 Shoval G et al. *JAMA Network Open.*

eTable 8. (cont.)

	Externalizing symptoms (standardized) > 1 SD									
	No AD/AP					Any AD/AP				
	No ADHD medication		Any ADHD medication			No ADHD medication		Any ADHD medication		
	n=391		*n=391*			*n=40*		*n=40*		
	Mean/ n	SD/%	Mean/ n	SD/%	*p*-value[b]	Mean/ n	SD/%	Mean/ n	SD/%	*p*-value[b]
**Age at assessment, years**	10.0	0.6	10.0	0.6	0.973	10.1	0.7	10.0	0.7	0.478
**Sex, female**	101	25.8%	103	26.3%	0.935	13	32.5%	13	32.5%	1.000
**White**	310	79.3%	309	79.0%	1.000	34	85.0%	36	90.0%	0.735
**Black**	95	24.3%	96	24.6%	1.000	6	15.0%	6	15.0%	1.000
**Asian**	12	3.1%	12	3.1%	1.000	1	2.5%	1	2.5%	1.000
**Hispanic**	52	13.3%	55	14.1%	0.835	4	10.0%	3	7.5%	1.000
**Parents' education, years**	16.3	2.4	16.4	2.4	0.455	17.2	2.1	17.4	1.4	0.736
**Parents married**	234	59.8%	230	58.8%	0.827	27	67.5%	24	60.0%	0.642
**Parents divorced/separated**	67	17.1%	72	18.4%	0.708	7	17.5%	9	22.5%	0.780
**Positive school involvement**	12.5	2.6	12.5	2.7	1.000	12.3	2.7	12.2	3.0	0.844
**Family conflict scale**	2.4	2.1	2.5	2.0	0.566	2.6	2.3	2.8	2.1	0.762
**Parental supervision**	4.2	0.6	4.2	0.6	0.628	4.3	0.6	4.3	0.7	0.973
**Weekend screen time**	5.9	4.3	6.0	4.3	0.960	5.1	4.4	4.7	3.3	0.595

[b] T-test and Chi-square comparisons for continuous and binary measures, respectively
Abbreviations: ADHD = Attention Deficit Hyperactivity Disorder; AD/AP = antidepressant or antipsychotic medication; SD = standard deviation; NA = not available.

© 2021 Shoval G et al. *JAMA Network Open.*

eTable 8. (cont.)

	Externalizing symptoms (standardized) > 2 SD									
	No AD/AP					Any AD/AP				
	No ADHD medication		Any ADHD medication			No ADHD medication		Any ADHD medication		
	n=221		*n=221*			*n=23*		*n=23*		
	Mean/ n	SD/%	Mean/ n	SD/%	*p*-value[b]	Mean/ n	SD/%	Mean/ n	SD/%	*p*-value[b]
**Age at assessment, years**	10.0	0.6	10.0	0.6	0.870	10.2	0.7	10.3	0.7	0.717
**Sex, female**	50	22.6%	51	23.1%	1.000	6	26.1%	5	21.7%	1.000
**White**	169	76.5%	170	76.9%	1.000	20	87.0%	18	78.3%	0.697
**Black**	57	25.8%	59	26.7%	0.914	4	17.4%	4	17.4%	1.000
**Asian**	11	5.0%	7	3.2%	0.470	0	0%	0	0%	NA
**Hispanic**	26	11.8%	25	11.3%	1.000	2	8.7%	2	8.7%	1.000
**Parents' education, years**	16.2	2.4	16.2	2.5	0.961	17.2	2.0	16.7	2.3	0.525
**Parents married**	131	59.3%	126	57.0%	0.700	15	65.2%	13	56.5%	0.763
**Parents divorced/separated**	40	18.1%	42	19.0%	0.903	5	21.7%	7	30.4%	0.737
**Positive school involvement**	12.1	2.6	12.3	2.8	0.423	12.1	2.8	11.4	2.9	0.415
**Family conflict scale**	2.5	2.1	2.5	2.1	1.000	2.6	2.4	2.4	2.1	0.791
**Parental supervision**	4.2	0.6	4.2	0.6	0.809	4.2	0.7	4.1	0.7	0.800
**Weekend screen time**	6.4	4.6	6.2	4.3	0.759	5.5	5.0	5.3	4.3	0.919

[b] T-test and Chi-square comparisons for continuous and binary measures, respectively
Abbreviations: ADHD = Attention Deficit Hyperactivity Disorder; AD/AP = antidepressant or antipsychotic medication; SD = standard deviation; NA = not available.

© 2021 Shoval G et al. *JAMA Network Open.*

eTable 8. (cont.)

	Externalizing symptoms (standardized) > 3 SD						
	No AD/AP					Any AD/AP	
	No ADHD medication		Any ADHD medication			No ADHD medication	Any ADHD medication
	n=35		*n=35*			*Total n=7*	*Total n=32*
	Mean/ n	SD/%	Mean/ n	SD/%	*p*-value[b]	**N/A[c]**	**N/A[c]**
**Age at assessment, years**	10.2	0.6	10.1	0.6	0.447		
**Sex, female**	3	8.6%	4	11.4%	1.000		
**White**	22	62.9%	26	74.3%	0.440		
**Black**	15	42.9%	14	40.0%	1.000		
**Asian**	1	2.9%	1	2.9%	1.000		
**Hispanic**	4	11.4%	5	14.3%	1.000		
**Parents' education, years**	15.2	2.6	15.9	2.7	0.239		
**Parents married**	14	40.0%	16	45.7%	0.809		
**Parents divorced/separated**	6	17.1%	5	14.3%	1.000		
**Positive school involvement**	12.5	2.7	12.3	2.9	0.731		
**Family conflict scale**	3.3	2.3	3.3	2.6	0.922		
**Parental supervision**	4.4	0.5	4.3	0.6	0.573		
**Weekend screen time**	7.2	5.7	6.4	3.6	0.498		

[b] T-test and Chi-square comparisons for continuous and binary measures, respectively
[c] Total participants in >3SD in the children receiving AD/AP included 32 participants of which only 7 participants did not take ADHD medication, therefore no matching was done on this group.
Abbreviations: ADHD = Attention Deficit Hyperactivity Disorder; AD/AP = antidepressant or antipsychotic medication; SD = standard deviation; NA = not available.

© 2021 Shoval G et al. *JAMA Network Open.*

eTable 9. Effect of Baseline Depression and Anxiety on Main Model Results

**Adjusted Model[a]**	**Cross sectional suicidality**					**1-year follow-up suicidality[b]**				
	B	SE	Wald	OR	*p*-value	B	SE	Wald	OR	*p*-value[c]
**Externalizing symptoms[d]**	0.272	0.03	81.047	1.313	<.001	0.193	0.066	8.449	1.213	0.004
**Any ADHD medication**	0.288	0.114	6.367	1.334	0.012	0.611	0.229	7.144	1.842	0.008
**Externalizing X ADHD meds[e]**	-0.236	0.09	6.973	0.789	0.008	-0.353	0.182	3.759	0.702	0.027
**Depression**	1.218	0.108	127.943	3.379	<.001	0.46	0.239	3.693	1.584	0.055
**Anxiety**	1.268	0.127	100.375	3.555	<.001	0.637	0.272	5.504	1.891	0.019

[a] Binary logistic regression model with suicidality as the dependent variable testing main effect and interaction of externalizing symptom count (standardized) and ADHD medication (binary variable) and co-varying for age, parents' education, marital status, race (White, Black, Asian, other), Hispanic ethnicity, whether the child was receiving antidepressants or antipsychotics, and two binary variables indicating history of depression or anxiety diagnoses, as determined in the K-SADS interview.
[b] Longitudinal model included current suicidality at the 1-year follow up assessment as the dependent variable, and co-varied for all variables as described above in addition to baseline suicidality and time between baseline and follow-up assessment.
[c] For the interaction model we used one-tailed test for the interaction effect based on anticipated direction of medication protective effect as observed in cross-sectional model findings, and based on preregistered hypothesis for protective effect of ADHD medication in children with high externalizing symptoms.
[d] The total count of externalizing symptoms was z-scored to allow easier interpretation of main effect, such that the obtained OR reflect a change in odds for a change in 1SD of externalizing symptoms.
[e] Interaction term was introduced in a separate model.
Abbreviations: ADHD = Attention Deficit Hyperactivity Disorder; externalizing = externalizing symptoms; meds = medications; SE = standard error; OR = odds ratio.

© 2021 Shoval G et al. *JAMA Network Open.*

eTable 10. Effect of Different Definitions of Externalizing Symptoms on Main Model Results

	Adjusted model[a]				
	B	SE	Wald	OR	*p*-value
**All ADHD, ODD and CD symptom sum[b]**	0.292	0.030	96.061	1.338	<.001
**Any ADHD medication**	0.301	0.112	7.270	1.351	0.007
**Externalizing X ADHD medication[c]**	-0.231	0.099	5.426	0.794	0.020
	B	SE	Wald	OR	*p*-value
**All ADHD symptom sum[b]**	0.234	0.030	60.433	1.264	<.001
**Any ADHD medication**	0.281	0.111	6.369	1.325	0.012
**Externalizing X ADHD medication[c]**	-0.158	0.107	2.196	0.854	0.138
	B	SE	Wald	OR	*p*-value
**Only hyperactive ADHD symptom sum[b]**	0.211	0.030	50.446	1.235	<.001
**Any ADHD medication**	0.271	0.112	5.901	1.311	0.015
**Externalizing X ADHD medication[c]**	-0.171	0.092	3.440	0.842	0.064

[a]Binary logistic regression model with suicidality as the dependent variable testing main effect and interaction of externalizing symptom count (standardized) and ADHD medication (binary variable) and co-varying for age, parents' education, marital status, race (White, Black, Asian, other) Hispanic ethnicity, and whether child was receiving antidepressants or antipsychotics. The model was repeated with different definitions of externalizing symptoms (all ADHD, ODD and CD symptoms; all ADHD symptoms; only hyperactive ADHD symptoms).
[b]To improve interpretability of externalizing symptoms main effect, ADHD medication variable was regressed out of the sum of externalizing symptoms (resulting in a z-score), hence that the obtained OR reflect a change in odds for a change in 1SD of externalizing symptoms.
[c]Interaction term was introduced in a separate model.
Abbreviations: ADHD = Attention Deficit Hyperactivity Disorder; ODD = Oppositional Defiant Disorder; CD = Conduct Disorder; externalizing = externalizing symptoms; SE = standard error; OR = odds ratio; SD = standard deviation.

© 2021 Shoval G et al. *JAMA Network Open.*

eTable 11. Sensitivity Analysis Using Suicidal Ideation (SI) as the Dependent Variable Instead ofSsuicidality (i.e., SI or Suicide Attempt) as in Main Model

**Adjusted Model**[a]	**Cross sectional suicidality**					**1-year follow-up suicidality**[b]				
	B	SE	Wald	OR	*p*-value	B	SE	Wald	OR	*p*-value[c]
**Externalizing symptoms**[d]	0.294	0.03	98.047	1.342	<.001	0.184	0.068	7.419	1.202	0.006
**Any ADHD medication**	0.319	0.112	8.096	1.375	0.004	0.541	0.236	5.27	1.719	0.022
**Externalizing X ADHD meds**[e]	-0.271	0.087	9.705	0.763	0.002	-0.313	0.187	2.805	0.731	0.047

[a] Binary logistic regression model with suicidality as the dependent variable testing main effect and interaction of externalizing symptom count (standardized) and ADHD medication (binary variable) and co-varying for age, parents' education, marital status, race (White, Black, Asian, other), Hispanic ethnicity, and whether child was receiving antidepressants or antipsychotics.
[b] Longitudinal model included current SI at the 1-year follow up assessment as the dependent variable, and co-varied for all variables as described above in addition to baseline SI and time between baseline and follow-up assessment.
[c] For the interaction model we used one-tailed test for the interaction effect based on anticipated direction of medication protective effect as observed in cross-sectional model findings, and based on preregistered hypothesis for protective effect of ADHD medication in children with high externalizing symptoms.
[d] To improve interpretability of externalizing symptoms main effect, ADHD medication variable was regressed out of the sum of externalizing symptoms (resulting in a z-score), hence that the obtained OR reflect a change in odds for a change in 1SD of externalizing symptoms.
[e] Interaction term was introduced in a separate model.
Abbreviations: ADHD = Attention Deficit Hyperactivity Disorder; meds = medications; externalizing = externalizing symptoms; SE = standard error; OR = odds ratio; SD = standard deviation.

© 2021 Shoval G et al. *JAMA Network Open.*

eTable 12. Sensitivity Analysis Using Parent-Report Suicidality as the Dependent Variable Instead of Child-Report Suicidality as in Main Model

	**Cross-sectional adjusted model[a]**				
	B	SE	Wald	OR	*P value*
**Externalizing symptoms[b]**	0.708	0.03	542.076	2.029	<.001
**Any ADHD medication**	0.851	0.108	61.895	2.342	<.001
**Externalizing X ADHD meds[c]**	-0.37	0.087	18.054	0.69	<.001

Note- Parent report of suicidality was not released by ABCD for the 1-year follow-up longitudinal assessment.

[a] Binary logistic regression model with suicidality as the dependent variable testing main effect and interaction of externalizing symptom count (standardized) and ADHD medication (binary variable) and co-varying for age, parents' education, marital status, race (White, Black, Asian, other), Hispanic ethnicity, and whether child was receiving antidepressants or antipsychotics.

[b] To improve interpretability of externalizing symptoms main effect, ADHD medication variable was regressed out of the sum of externalizing symptoms (resulting in a z-score), hence that the obtained OR reflect a change in odds for a change in 1SD of externalizing symptoms.

[c] Interaction term was introduced in a separate model.

Abbreviations: ADHD = Attention Deficit Hyperactivity Disorder; meds = medications; externalizing = externalizing symptoms; SE = standard error; OR = odds ratio; SD = standard deviation.

© 2021 Shoval G et al. *JAMA Network Open.*

eTable 13. Imputed Data Sensitivity Analysis

**Cross sectional models**	**Adjusted Model[a]**					**Adjusted Model[b]**				
	B	SE	Wald	OR	*p*-value	B	SE	Wald	OR	*p*-value
**Externalizing symptoms[c]**	0.296	0.029	104.379	1.345	<.001	0.216	0.03	51.471	1.241	<.001
**Any ADHD medication**	0.269	0.111	5.9	1.309	0.015	0.117	0.113	1.061	1.124	0.303
**Externalizing X ADHD meds[d]**	-0.259	0.086	9.207	0.771	0.002	-0.194	0.088	4.802	0.824	0.014
**1-year longitudinal models[e]**	**Adjusted Model[a]**					**Adjusted Model[b]**				
	B	SE	Wald	OR	*p*-value[f]	B	SE	Wald	OR	*p*-value[f]
**Externalizing symptoms[c]**	0.191	.065	8.624	1.211	0.003	0.111	0.067	2.772	1.117	0.096
**Any ADHD medication**	0.555	0.228	5.942	1.742	0.015	0.418	0.228	3.35	1.519	0.067
**Externalizing X ADHD meds[c]**	-0.344	0.18	3.658	0.709	0.028	-0.255	0.183	1.942	0.775	0.082

[a] Binary logistic regression model with suicidality as the dependent variable testing main effect and interaction of externalizing symptom count (standardized) and ADHD medication (binary variable) and co-varying for age, parents' education, marital status, race (White, Black, Asian, other), Hispanic ethnicity, and whether child was receiving antidepressants or antipsychotics.
[b] Model 2 is similar to model 1 in addition to the four risk and protective factors described previously: [Janiri et al. 2020]: family conflict, weekend screen time, parental supervision and positive school involvement.
[c] To improve interpretability of externalizing symptoms main effect, ADHD medication variable was regressed out of the sum of externalizing symptoms (resulting in a z-score), hence that the obtained OR reflect a change in odds for a change in 1SD of externalizing symptoms.
[d] Interaction term was introduced in a separate model.
[e] Longitudinal model included current suicidality at the 1-year follow up assessment as the dependent variable, and co-varied for all variables as described above in addition to baseline suicidality and time between baseline and follow-up assessment.
[f] For the interaction model we used one-tailed test for the interaction effect based on anticipated direction of medication protective effect as observed in cross-sectional model findings, and based on preregistered hypothesis for protective effect of ADHD medication in children with high externalizing symptoms.
Abbreviations: ADHD = Attention Deficit Hyperactivity Disorder; meds = medications; externalizing = externalizing symptoms; SE = standard error; OR = odds ratio; SD = standard deviation.

© 2021 Shoval G et al. *JAMA Network Open.*

eTable 14. Family Relatedness Sensitivity Analysis

Adjusted Model[a]	No related children (n=7 582)					Only one child from family (n=9 468)				
	B	SE	Wald	OR	*p*-value	B	SE	Wald	OR	*p*-value
**Externalizing symptoms[b]**	0.300	0.037	66.187	1.349	<.001	0.301	0.033	84.409	1.352	<.001
**Any ADHD medication**	0.284	0.138	4.232	1.328	0.040	0.223	0.126	3.132	1.250	0.077
**Externalizing X ADHD meds[c]**	-0.364	0.108	11.263	0.695	0.001	-0.263	0.100	6.964	0.769	0.008

[a] Binary logistic regression model with suicidality as the dependent variable testing main effect and interaction of externalizing symptom count (standardized) and ADHD medication (binary variable) and co-varying for age, parents' education, marital status, race (White, Black, Asian, other), Hispanic ethnicity, and whether child was receiving antidepressants or antipsychotics.
[b] To improve interpretability of externalizing symptoms main effect, ADHD medication variable was regressed out of the sum of externalizing symptoms (resulting in a z-score), hence that the obtained OR reflect a change in odds for a change in 1SD of externalizing symptoms.
[c] Interaction term was introduced in a separate model.
The main model was repeated twice to account for the sets of related children in the total sample.
In the first, all related children were excluded. In the second, one child from each family was randomly selected for inclusion.
Abbreviations: ADHD = Attention Deficit Hyperactivity Disorder; meds=medications; externalizing = externalizing symptoms; SE = standard error; OR = odds ratio; SD = standard deviation.

© 2021 Shoval G et al. *JAMA Network Open.*

eTable 15. Exploratory Analyses of Different ADHD Medication Class

	**MPH (n=541, 4.6% of sample)**						
	B	SE	Wald	OR	Low limit 95% CI	Upper limit 95% CI	*p*-value
**Ext. symp.**[a]	0.291	0.030	96.499	1.338	1.262	1.417	<.001
**ADHD med**	0.265	0.140	3.559	1.303	0.990	1.716	0.059
**Ext. by meds**[b]	-0.416	0.112	13.803	0.660	0.530	0.822	<.001
	**Amphetamine (n=363, 3.1% of sample)**						
	B	SE	Wald	OR	Low limit 95% CI	Upper limit 95% CI	*p*-value
**Ext. symp.** [a]	0.290	0.030	95.714	1.336	1.261	1.416	<.001
**ADHD med**	0.115	0.178	0.421	1.122	0.792	1.590	0.516
**Ext. by meds**[b]	-0.001	0.141	0	0.999	0.757	1.317	0.994
	**Alpha-agonists (n=243, 2% of sample)**						
	B	SE	Wald	OR	Low limit 95% CI	Upper limit 95% CI	*p*-value
**Ext. symp.** [a]	0.290	0.030	95.864	1.337	1.261	1.417	<.001
**ADHD med**	-0.200	0.215	0.866	0.819	0.538	1.247	0.352
**Ext. by meds**[b]	-0.069	0.175	0.155	0.933	0.662	1.315	0.694
	**Atomoxetine (n=47, 0.4% of sample)**						
	B	SE	Wald	OR	Low limit 95% CI	Upper limit 95% CI	*p*-value
**Ext. symp.** [a]	0.290	0.030	95.642	1.336	1.261	1.416	<.001
**ADHD med**	0.531	0.394	1.823	1.701	0.787	3.681	0.177
**Ext. by meds**[b]	-0.541	0.345	2.465	0.582	0.296	1.144	0.116

Results were derived from binary logistic regression models with suicidality as the dependent variable testing main effect and interaction of externalizing symptom count (standardized) and ADHD medication (binary variable) and co-varying for age, parents' education, marital status, race (White, Black, Asian, other), Hispanic ethnicity, and whether child was receiving antidepressants or antipsychotics.
The model was run independently for each of the 4 ADHD medication classes, which were grouped into one variable for the main analysis.
[a] To improve interpretability of externalizing symptoms main effect, ADHD medication variable was regressed out of the sum of externalizing symptoms (resulting in a z-score), hence that the obtained OR reflect a change in odds for a change in 1SD of externalizing symptoms.
[b] Interaction term was introduced in a separate model.
Abbreviations: ADHD = Attention Deficit Hyperactivity Disorder; MPH = methylphenidate derivative; Ext. symp. = externalizing symptoms (standardized); meds=medications; SE = standard error; OR = odds ratio; CI = confidence interval; SD = standard deviation.

© 2021 Shoval G et al. *JAMA Network Open.*

eFigure. Moderating Effect of ADHD Medication Use at Baseline on the Relationship Between Baseline Externalizing Symptoms and Suicidality in 1-Year Follow-up Assessment

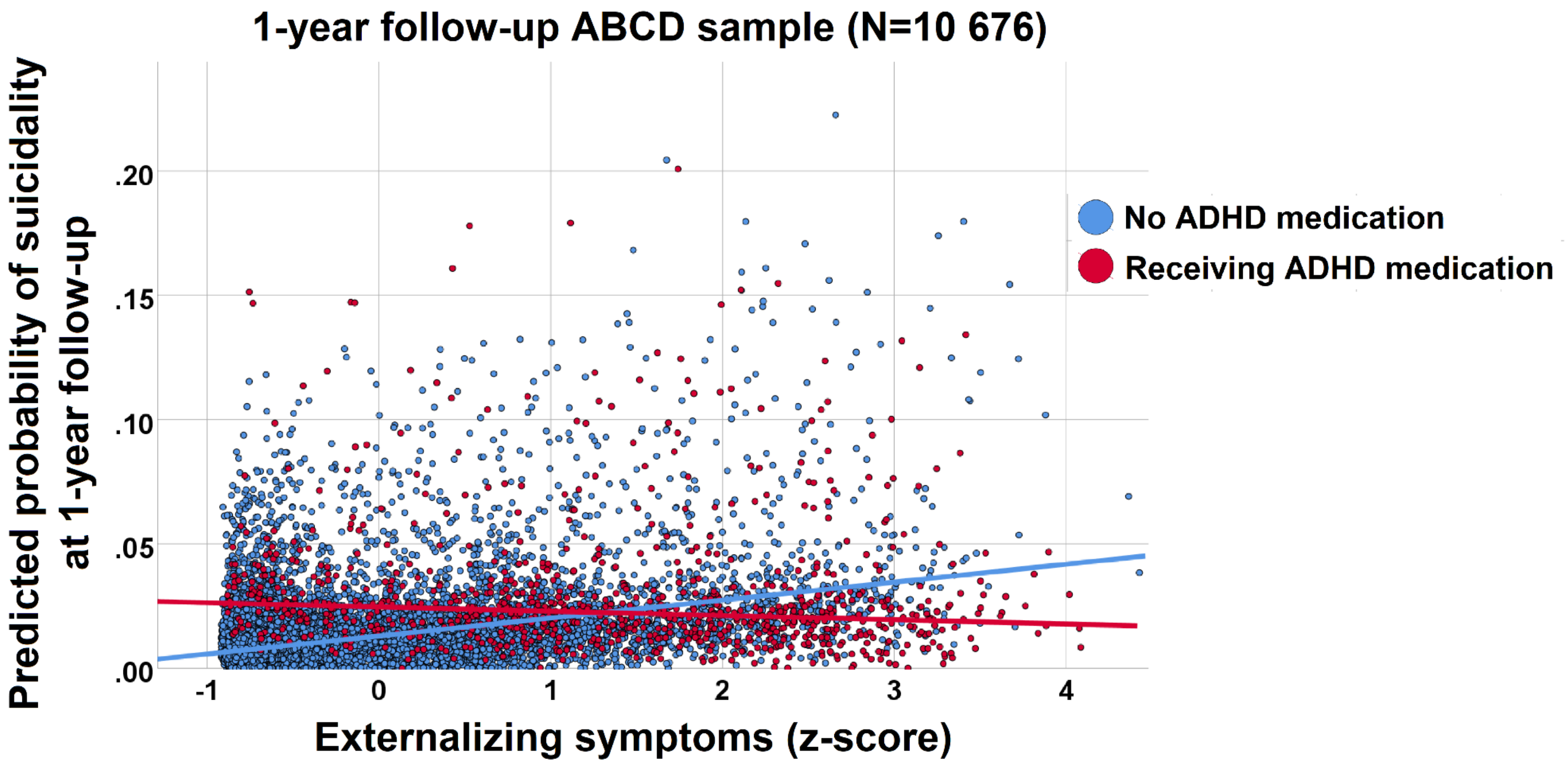

Caption: Scatter plots and linear regression lines visualizing predicted probabilities of current suicidality at 1-year follow-up assessment among all ABCD participants with longitudinal data (n=10 676).
Medication treatment and externalizing symptoms (hyperactivity ADHD symptoms, ODD and CD symptoms) were captured at baseline assessment.
Abbreviations: ADHD = Attention Deficit Hyperactivity Disorder; ODD = Oppositional Defiant Disorder; CD = Conduct Disorder

© 2021 Shoval G et al. *JAMA Network Open.*
